# Molecular regulatory mechanism of key LncRNAs in subclinical mastitic cows with folic acid supplementation

**DOI:** 10.1186/s12864-023-09466-3

**Published:** 2023-08-17

**Authors:** Xueqin Liu, Siyuan Mi, Wenlong Li, Jinning Zhang, Serafino M. A. Augustino, Zhichao Zhang, Ruiqiang Zhang, Wei Xiao, Ying Yu

**Affiliations:** 1 Key Laboratory of Animal Genetics, Breeding and Reproduction, Ministry of Agriculture, National Engineering Laboratory for Animal Breeding, College of Animal Science and Technology, China Agricultural University, Beijing, 100193 China; 2 School of Natural Resources and Environmental Studies, University of Juba, P. O. Box 82, Juba, South Sudan; 3 Beijing Animal Husbandry Station, Beijing, 100029 China

**Keywords:** Subclinical mastitis, Folic acid supplementation, LncRNA, Chinese holstein cows

## Abstract

**Background:**

Folic acid is a water-soluble B vitamin (B9), which is closely related to the body’s immune and other metabolic pathways. The folic acid synthesized by rumen microbes has been unable to meet the needs of high-yielding dairy cows. The incidence rate of subclinical mastitis in dairy herds worldwide ranged between 25%~65% with no obvious symptoms, but it significantly causes a decrease in lactation and milk quality. Therefore, this study aims at exploring the effects of folic acid supplementation on the expression profile of lncRNAs, exploring the molecular mechanism by which lncRNAs regulate immunity in subclinical mastitic dairy cows.

**Results:**

The analysis identified a total of 4384 lncRNA transcripts. Subsequently, differentially expressed lncRNAs in the comparison of two groups (SF vs. SC, HF vs. HC) were identified to be 84 and 55 respectively. Furthermore, the weighted gene co-expression network analysis (WGCNA) and the KEGG enrichment analysis result showed that folic acid supplementation affects inflammation and immune response-related pathways. The two groups have few pathways in common. One important lncRNA MSTRG.11108.1 and its target genes (*ICAM1*, *CCL3*, *CCL4*, etc.) were involved in immune-related pathways. Finally, through integrated analysis of lncRNAs with GWAS data and animal QTL database, we found that differential lncRNA and its target genes could be significantly enriched in SNPs and QTLs related to somatic cell count (SCC) and mastitis, such as MSTRG.11108.1 and its target gene *ICAM1*, *CXCL3*, *GRO1*.

**Conclusions:**

For subclinical mastitic cows, folic acid supplementation can significantly affect the expression of immune-related pathway genes such as *ICAM1* by regulating lncRNAs MSTRG.11108.1, thereby affecting related immune phenotypes. Our findings laid a ground foundation for theoretical and practical application for feeding folic acid supplementation in subclinical mastitic cows.

**Supplementary Information:**

The online version contains supplementary material available at 10.1186/s12864-023-09466-3.

## Background

Mastitis in cows refers to a kind of mammary gland inflammation caused by the stimulation of various physical and chemical factors and microorganisms (such as *Staphylococcus aureus*) [1]. As a common disease, mastitis in cows not only reduces the yield and quality of milk but also threatens animal health and welfare [2]. Cow mastitis can be divided into subclinical mastitis and clinical mastitis. Subclinical mastitis in dairy cows has no clinical symptoms and is not easily detected. A continuous infection will reduce the production performance of dairy cows and may develop into clinical mastitis, increasing the cost of treatment, and resulting in huge economic losses for the dairy cattle industry [3]. According to previous reports, the global incidence of subclinical mastitis in dairy herds is about 25%~65% [4]. Mastitis in dairy cows has low heritability, so conventional breeding strategies are difficult to achieve satisfying results [5, 6]. In the past decade, although Genome-Wide Association Studies (GWAS) had identified many SNPs related to mastitic resistance, most of these SNPs are located in non-coding regions [7].

Long noncoding RNAs (lncRNAs) are a class of non-coding RNAs with transcript lengths ≥ 200 nt, and they play a crucial role in regulating gene expression. Studies have shown that lncRNAs have many biological functions, such as participating in cell proliferation [8, 9], differentiation [10, 11], apoptosis [12, 13], promoting myoblast differentiation and injury-induced muscle regeneration [14], fat deposition [15], lactation [16], reproduction [17], immunity [18] and many other life processes. Studies on humans and model animals have shown that lncRNA is involved in mammalian mammary gland development and regulation of lactation processes [16, 19]. A previous study found that overexpression of lncRNA ROR can increase the self-renewal of breast stem cells. Through further research on its function, the results showed that lncRNA ROR plays a key role in maintaining the normal stem cell subpopulation of breast epithelial cells [20]. Due to the late start of lncRNA research in domestic animals, there are few research reports on lncRNA regulating cow mammary gland development and susceptibility to mastitis.

The health status of cow’s mammary gland is easily affected by the environment, such as the infection of pathogens, the management of pasture, and the conditions of feeding [21]. Folic acid is a water-soluble B vitamin (B9). As a substance that affects DNA synthesis and participates in the methionine cycle, folic acid is involved in metabolic pathways such as cell proliferation and milk protein synthesis [22]. It is essential for maintaining the body’s normal life activities. With the gradual in-depth research of folic acid in some congenital malformations, cardiovascular diseases, and tumours [23–25], the importance of folic acid has gradually been recognized. As a methyl donor [22], folic acid plays an important role in the control of gene expression and stability through its role in DNA and histone methylation. Many current research results showed that folic acid not only improves milk quality, but supplementation with appropriate folic acid can effectively reduce the secretion of certain inflammatory factors in the body, thereby reducing inflammation and enhancing the body’s immunity [26, 27]. However, the impact of folic acid on cows with subclinical mastitis is rarely reported. Many studies have shown that folic acid has a significant effect on immunity [28–30], but the molecular mechanism underlying immunity regulation by lncRNA in mastitic cows fed with supplemented folic acid remains largely unclear.

Therefore to address this issue, and to explore the molecular mechanism by which lncRNA regulates immunity in both subclinical mastitic and healthy dairy cows fed with a diet supplemented with folic acids, we carried out the analysis of the expression profiles of mRNAs and lncRNAs in subclinical mastitic and healthy dairy cows fed with supplemented folic acids using RNA sequencing. The findings of this study provide a theoretical basis and a practical foundation for mammary health. Therefore, in general, the study provides a foundation for future studies to build on these results and verify the regulatory effects of lncRNAs in subclinical mastitic dairy cows.

## Results

### Overview of the RNA-sequencing data and identification of putative LncRNAs

To investigate the roles of folic acid on subclinical mastitic cows, the blood buffy coat of 14 Holstein dairy cows was collected for transcriptome sequencing. The Illumina Novaseq 6000 platform was used to perform RNA-seq, and 150 bp paired-end reads were generated. The average number of raw data was 65.2 million for 14 RNA-seq libraries. After filtering the raw reads (removing low-quality sequences and adaptor sequences), an average of 62.9 million paired-end reads (range: 55 ~ 80 million) for the mRNAs and lncRNAs were obtained from each library. More than 93% of clean reads were mapped to the bovine genome (Ensembl Bos taurus ARS-UCD1.2) using HISAT2. The details of sequencing data are shown in Table S1. In our previous research, the above RNA sequencing data has been verified on selected genes by RT-qPCR.

**Fig. 1:**
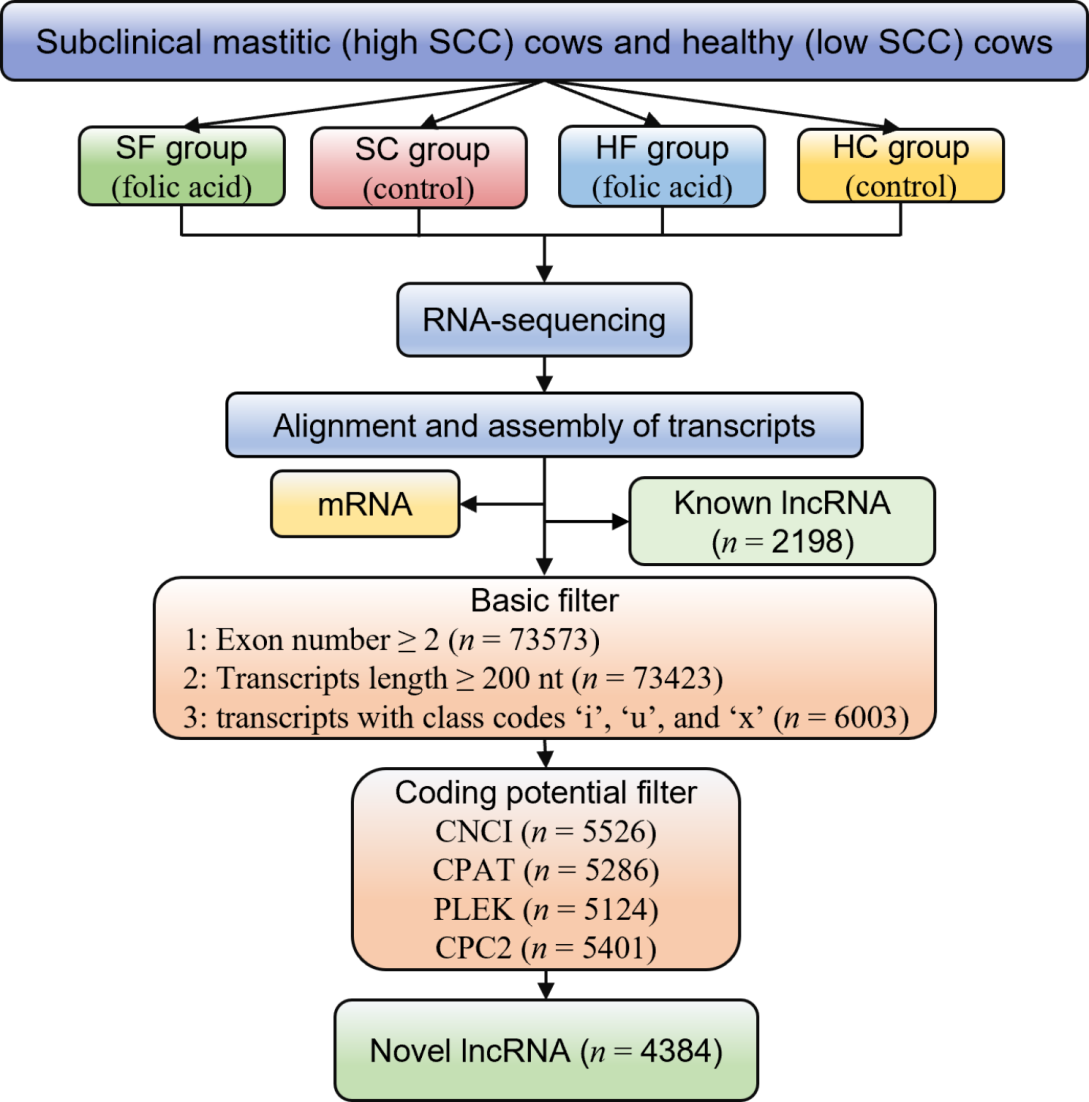
LncRNA analysis pipeline. Subclinical mastitis supplementary feeding group (SF), Subclinical mastitis control group (SC), Health supplementary feeding group (HF) , and Health control group (HC). The transcripts annotated as lncRNA in Ensembl are known lncRNA. While the transcripts which can not be annotated as lncRNA but are retained after the basic filter and coding potential filter are identified as novel lncRNA. *n* represents the number of transcripts

**Fig. 2:**
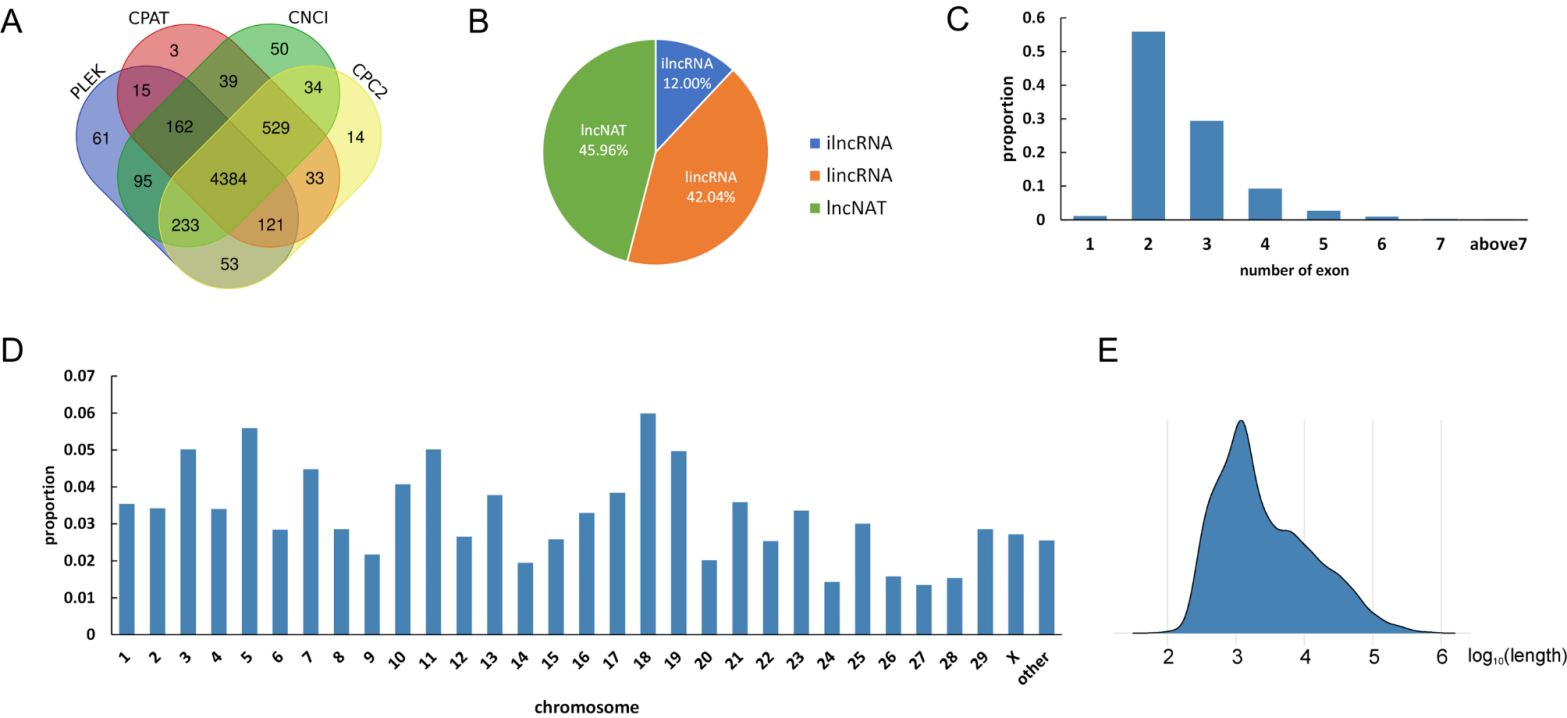
Features of lncRNA. (**A**) The Venn diagram of lncRNAs identified by four coding-potential prediction software. (**B**) Classification of novel lncRNA. (**C**) The number of exons of the identified lncRNA transcripts. (**D**) Distribution of identified lncRNA transcripts across chromosomes. (**E**) Length distribution of the lncRNA transcripts

To identify reliable putative lncRNAs from assembled transcripts, we employed a stringent pipeline using different filter criteria (Fig. 1). In this study, 6582 lncRNA transcripts were obtained, of which 2,198 were known and 4,384 were novel (Fig. 2A). Among them (Fig. 2B), 42.04% were lincRNAs (intergenic lncRNAs), 12.00% were ilncRNAs (intronic lncRNAs), and 45.96% were lncNAT (antisense lncRNAs). More than half of the lncRNA transcripts (55.9%) have two exons (Fig. 2C). Seventy-eight lncRNAs have only one exon, and all are known lncRNA. As shown in Fig. 2D, most of the identified lncRNAs are located on autosomal chromosomes, with more transcripts found in Chr18 (394) and Chr5 (368). The genomic coordinates of the lncRNA transcripts identified in this study are summarized in Table S2. The length of the lncRNAs was mainly between 200 and 1,500 nt (84.40%, Fig. 2E).

### Folic acid supplementation affects inflammation and immune response-related pathways

**Fig. 3:**
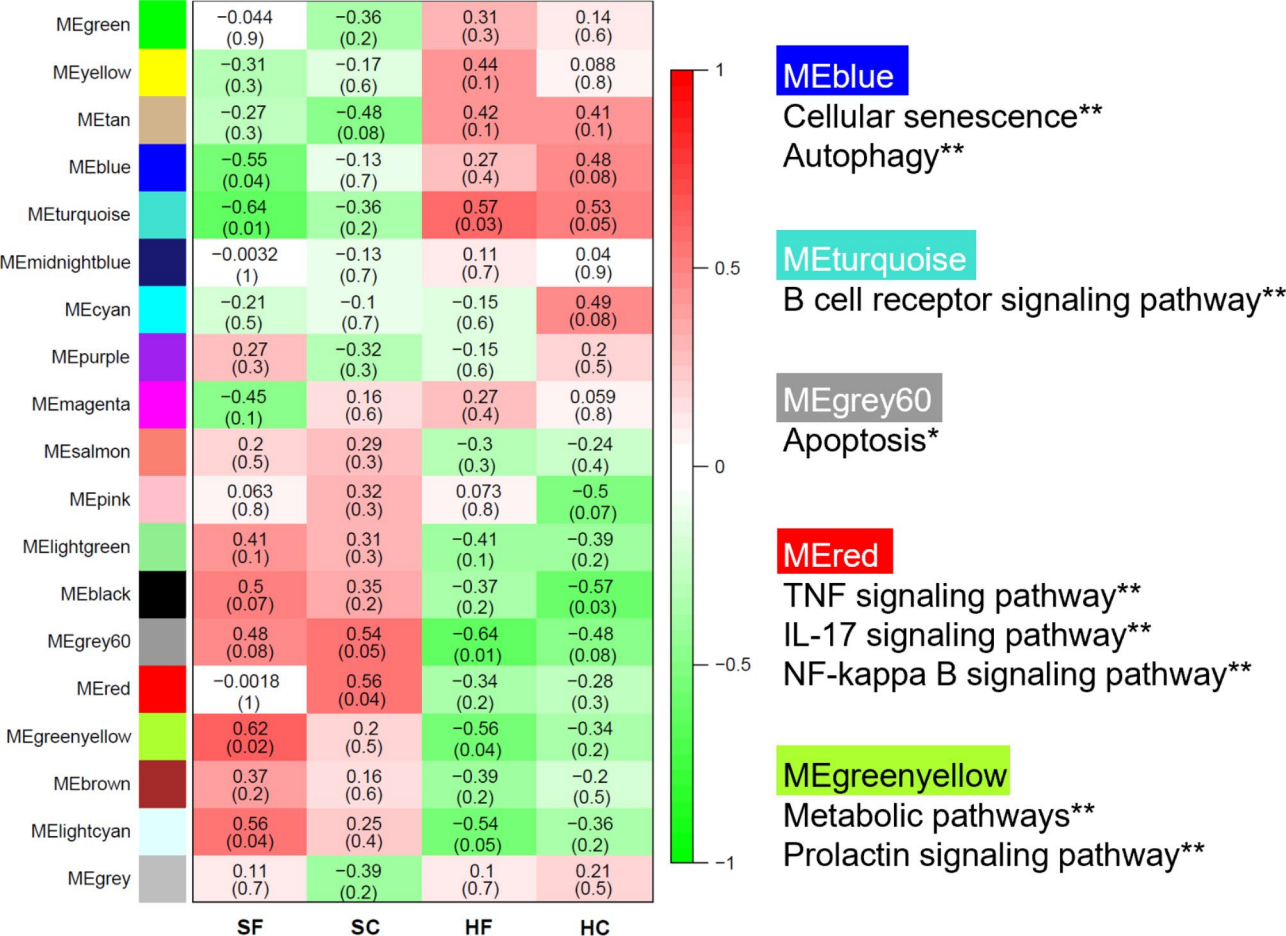
GNMs and phenotype association analysis and pathway enrichment result of genes in significant GNMs. The co-expressed genes within the WGCNA were identified as gene network modules (GNMs). Red represents positive correlations, green represents negative correlations, and the darker the color, the stronger the correlation. Each row represents a module, each column represents a group, and the color represents the correlation of module characteristic gene (ME) and group phenotype. *, 0.01 < *P* < 0.05. **, *P* < 0.01

The transcriptome data were analyzed, then 15,971 genes were obtained and used for the Weighted Gene Co-expression Network Analysis (WGCNA). In order to make the network close to the scale-free distribution, it is necessary to select *R*^*2*^ suitable for the value of parameter *β*. Theoretically, the closer *R*^*2*^ is to 1, the more the model conforms to the scale-free distribution. In this study, *R*^*2*^ > 0.9 was taken as an example, and it was found that *β* = 7 is more reasonable as a soft threshold for constructing a co-expression module. A total of 7 modules are closely related to the target physiological process (*P* < 0.05). Finally, 5 target modules were selected, namely “blue” (r = -0.55, *P* = 0.04), “turquoise” (r = -0.64, *P* = 0.01), “grey60” (r = -0.64, *P* = 0.01), “red” (r = 0.56, *P* = 0.04), and “green-yellow” (r = 0.62, *P* = 0.02). KEGG analysis results showed that the genes of these significant modules are mainly involved in immune-related pathways such as the B cell receptor signalling pathway, NF-kappa B signalling pathway, and Autophagy (Fig. 3).

### Differential expression profile of mRNA and lncRNA after folic acid supplementation

**Fig. 4:**
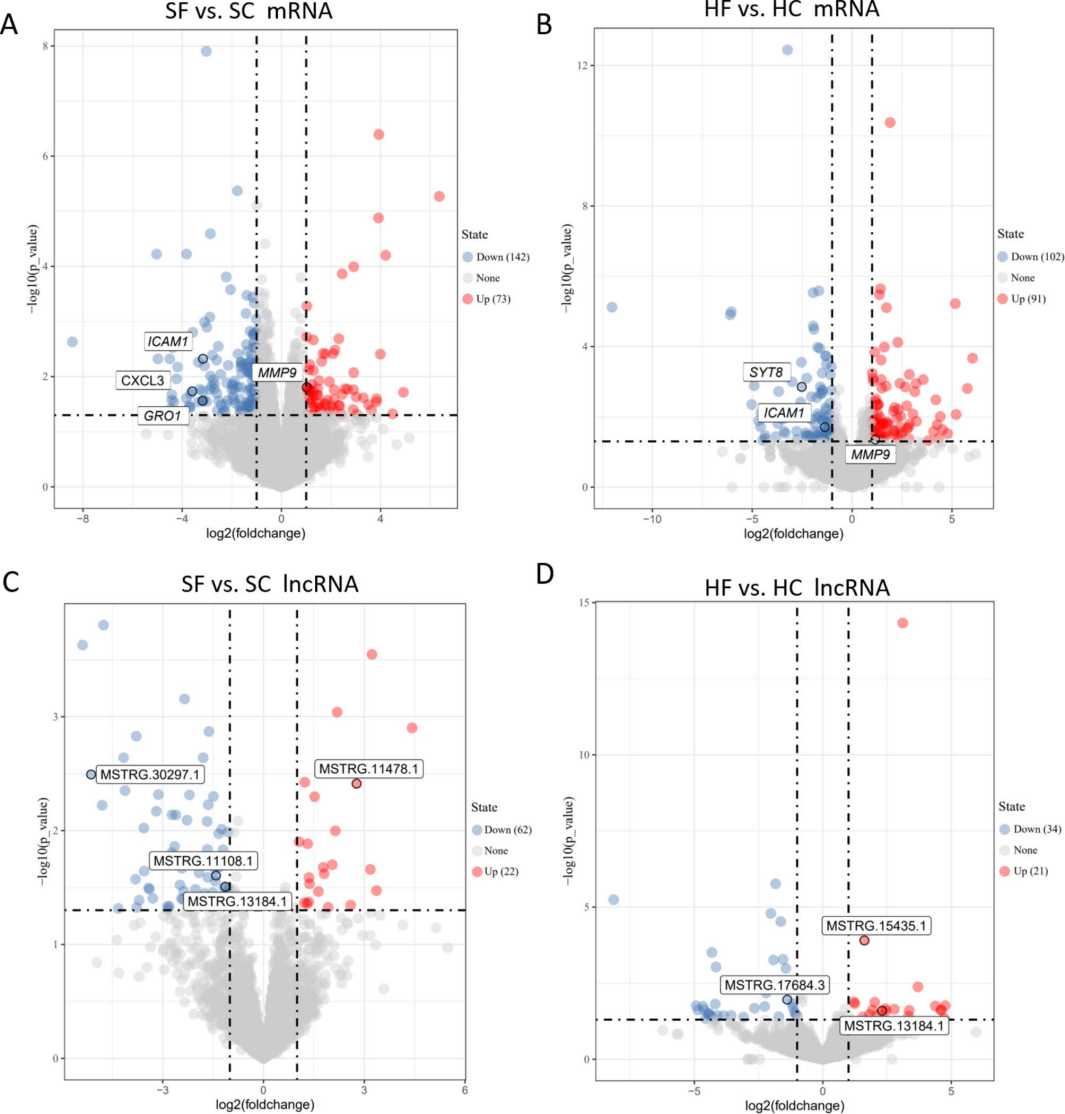
The volcano plots of the differentially expressed mRNAs and lncRNAs [log2(fold change) ≥ 1, *P*-value < 0.05]. Differentially expressed mRNAs in (**A**) SF vs. SC, (**B**) HF vs. HC; Differentially expressed lnRNAs in (**C**) SF vs. SC, (**D**) HF vs. HC. Red and blue colors indicate significantly up-and down-regulation, respectively. Grey indicates no significant regulation

In order to determine the molecular regulatory mechanism of folic acid affecting the immunity of subclinical mastitic cows, the expression level changes of lncRNAs and mRNAs were detected in the blood buffy coat of the four groups. In this study, log_2_|fold change| > 1 and *P* < 0.05 were set as the criteria of differential expression. A total of 215 and 193 mRNAs were differentially expressed (DE mRNAs) in SF vs. SC (Fig. 4A and Table S3), HF vs. HC (Fig. 4B and Table S4), respectively. There are 15 DE mRNAs shared in the two groups (Table 1).

**Table 1:** The differentially expressed mRNAs shared in both SF vs. SC and HF vs. HC

ID	SF vs. SC			HF vs. HC	
	log2FoldChange	pvalue		log2FoldChange	pvalue
ENSBTAG00000020676	1.02141	0.015537		1.159553	0.044376
ENSBTAG00000033278	1.015077	0.016469		-1.2502	0.013512
ENSBTAG00000046900	1.288896	0.002164		-1.20589	0.028207
ENSBTAG00000049252	1.456705	0.021729		-2.04834	0.038247
ENSBTAG00000020657	1.181837	0.030384		-1.56274	0.026203
ENSBTAG00000008102	-3.03056	1.25E-08		3.557471	0.000873
ENSBTAG00000004574	-1.29523	0.029759		1.617777	0.036439
ENSBTAG00000016713	-1.24634	0.004976		1.390926	0.048112
ENSBTAG00000022807	-1.11625	0.016776		2.249306	0.010536
ENSBTAG00000021672	-1.7669	0.015431		-2.57396	0.003803
ENSBTAG00000010303	-3.1629	0.004747		-1.36418	0.019584
ENSBTAG00000050334	-2.23789	0.031922		-2.32905	0.028488
ENSBTAG00000014861	-1.1557	0.00036		-1.05887	0.036691
ENSBTAG00000006383	-3.57022	0.00157		-2.7064	0.005605
ENSBTAG00000042458	-1.67516	0.025334		-3.67905	0.001893

Meanwhile, 84 differentially expressed lncRNAs (DE lncRNAs) were identified in SF vs. SC, including 22 up-regulated and 62 down-regulated lncRNA (Fig. 4C and Table S5). In the HF vs. HC, there are 55 DE lncRNAs (including 21 up-regulated and 34 down-regulated), which is less compared to that of SF vs. SC (Fig. 4D and Table S6). As shown in the Table 2, there are only seven DE lncRNAs shared between the two groups (SF vs. SC and HF vs. HC). In addition, the rlog-normalized read counts of the DE mRNAs and DE lncRNAs in the two comparisons (SF vs. SC and HF vs. HC) were analyzed by hierarchical clustering. As shown in the clustering heatmap (Figure S1), the difference in the expression patterns of DE mRNAs and DE lncRNAs in the two groups was obvious.

**Table 2:** The differentially expressed lncRNAs shared in both SF vs. SC and HF vs. HC

ID	SF vs. SC			HF vs. HC	
	log2FoldChange	pvalue		log2FoldChange	pvalue
MSTRG.6499.1	-1.62164	0.001348		2.462827	0.025799
MSTRG.13184.1	-1.13768	0.031056		2.304492	0.025538
MSTRG.15334.1	-1.30368	0.020797		3.109891	4.68E-15
MSTRG.7445.4	-2.20322	0.004863		-1.71373	0.040012
MSTRG.7446.1	-1.64523	0.00593		-1.91793	0.000547
MSTRG.32396.1	-4.76054	0.000157		-4.31963	0.000309
MSTRG.7445.5	-1.66949	0.014645		-2.26355	0.018517

### The effects of folic acid supplementation in subclinical mastitic and healthy cows are differnt through different regulatory mechanisms

**Fig. 5:**
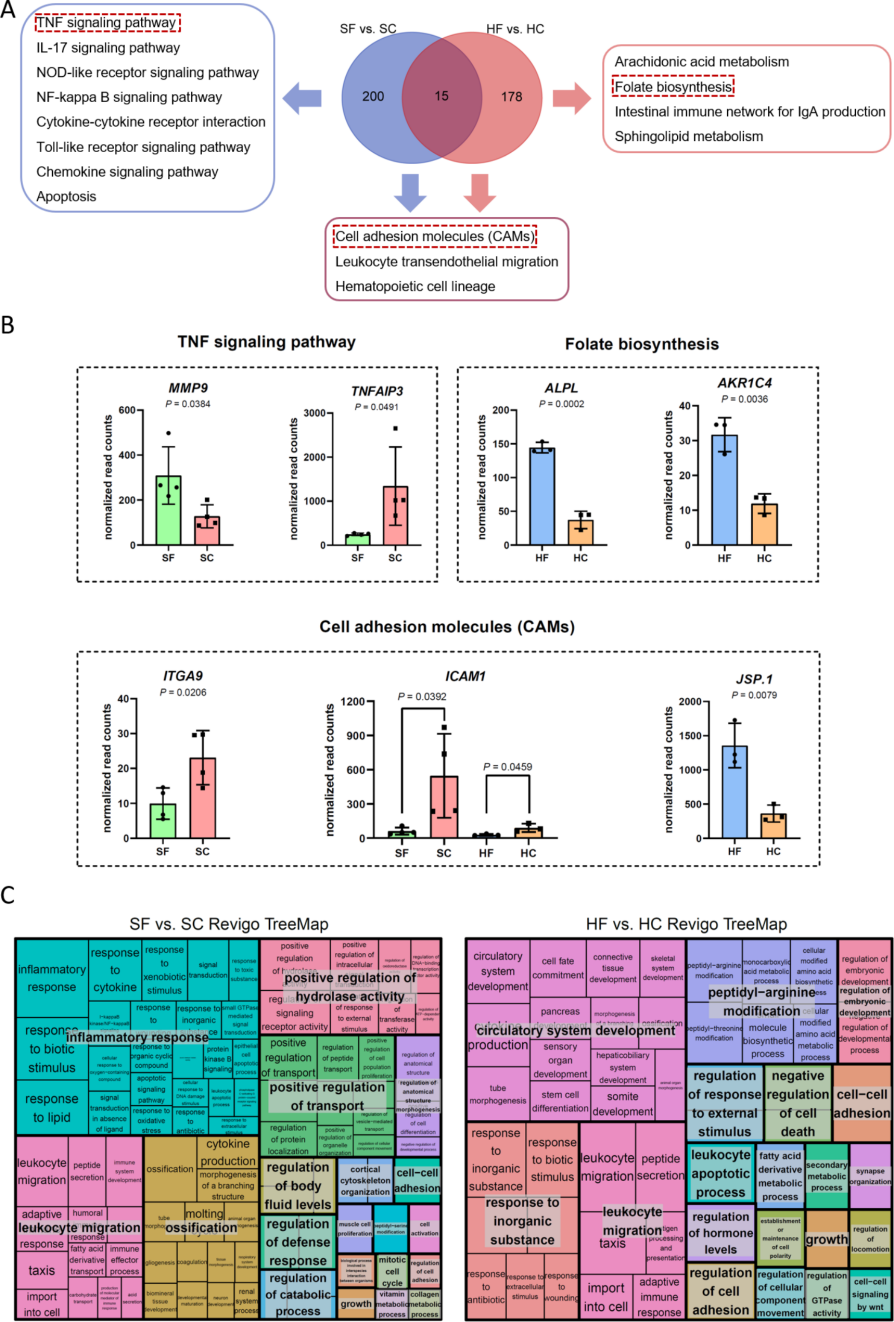
KEGG pathway and GO term enrichment of DE mRNAs. (**A**) Specific or shared KEGG pathway in SF vs. SC and HF vs. HC. (**B**) Expression differences of the DEGs involved in the enrichment of the TNF signaling pathway, folate biosynthesis pathway and cell adhesion molecules pathway. (**C**) Treemap showed significant BP-GO terms of DEGs in SF vs. SC and HF vs. HC (*P* < 0.05)

KEGG enrichment analysis was performed on the DE mRNAs in the comparison of both SF vs. SC, and HF vs. HC. A total of 53 pathways were significantly enriched (*P* < 0.05) (Table S7), in the comparison of SF vs. SC, most of these pathways are related to immune or inflammatory reactions. On the other hand, a total of 24 pathways were significantly enriched in the comparison of HF vs. HC (*P* < 0.05) (Table S8). Among these pathways, the same immune-related pathways are shared between SF vs. SC and HF vs. HC, such as cell adhesion molecules (CAMs), leukocyte transendothelial migration, hematopoietic cell lineage, etc. (Fig. 5A). In addition, the pathways related to metabolisms, such as Sphingolipid metabolism, Arachidonic acid metabolism, and Folate biosynthesis, were enriched in HF vs. HC. The unique and shared pathways in the two comparisons SF vs. SC and HF vs. HC were selected respectively. The DE mRNAs *MMP9* and *TNFAIP3* were enriched in the TNF signaling pathway, and the DE mRNAs *ALPL* and *AKR1C4* were enriched in the folate biosynthesis pathway. The shared pathway cell adhesion molecules (CAMs) was enriched by DE mRNAs such as *ITGA9*, *ICAM* and *JSP.1* (Fig. 5B). This study concentrates mainly on the biological process (BP). Therefore, the BP terms significantly enriched by DEGs are shown in Fig. 5C. The functional enrichment analysis results showed that after folic acid supplementation, the immune response in the peripheral blood leukocytes of subclinical mastitic cows and healthy cows is activated. Thus, the shared and different regulatory pathways between subclinical mastitic cows and healthy cows were identified.

### LncRNA regulates gene expression through cis and trans-acting mechanisms

**Fig. 6:**
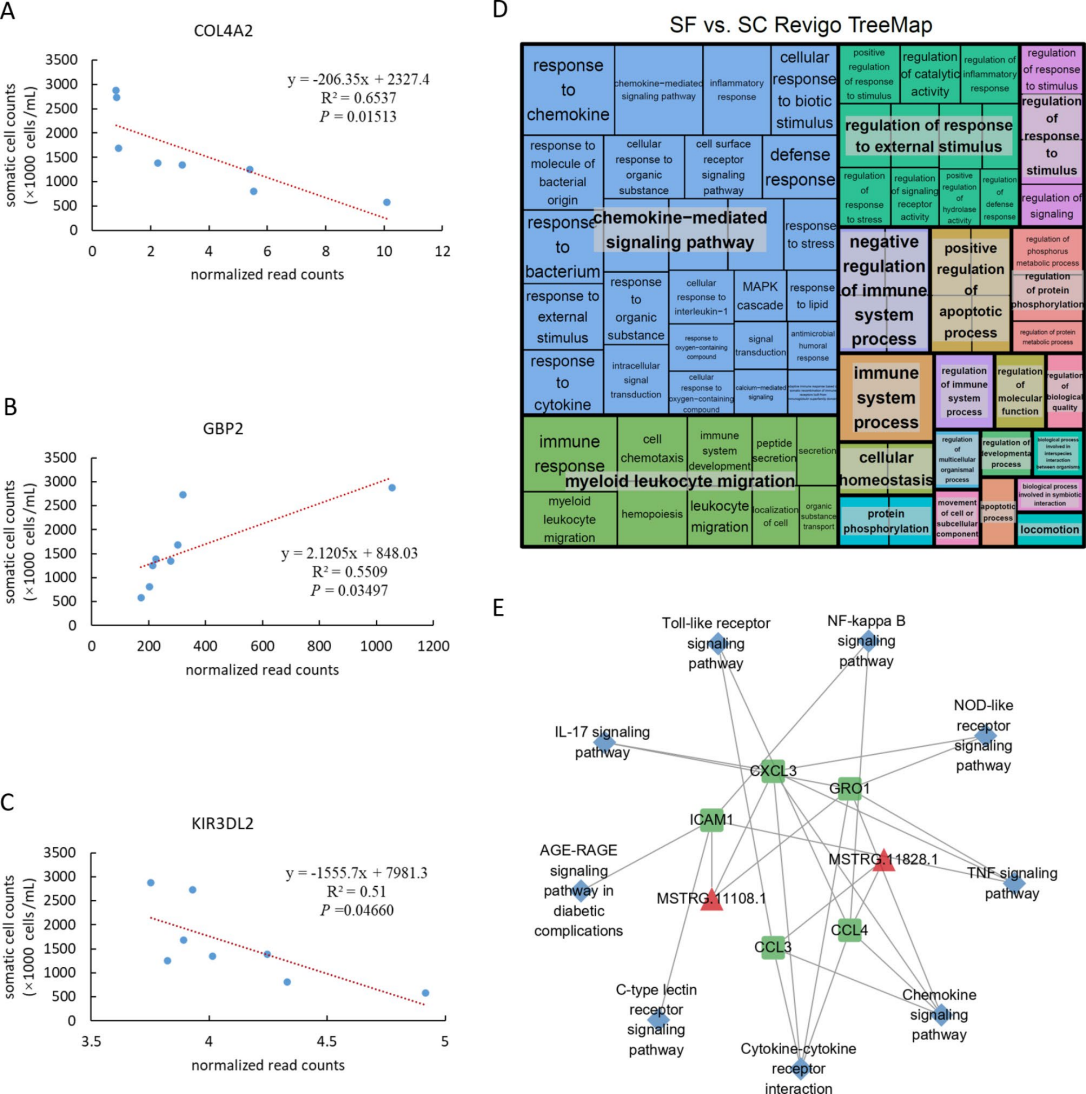
Functional analysis of DE lncRNAs and their target gene in the comparison of SF vs. SC. The linear regression between somatic cell counts (SCC) and (**A**) *COL4A2* (target gene of DE lncRNA MSTRG.5075.2), (**B**) *GBP2* (target gene of DE lncRNA MSTRG.22632.1), (**C**) *KIR3DL2* (target gene of DE lncRNA MSTRG.11478.1), respectively. (**D**) Treemap shows a significant GO term of DE lncRNA target genes. (**E**) The relationship of lncRNA-mRNA-pathway. Red triangles represent differentially expressed lncRNAs; green round rectangles represent differentially expressed targets; blue diamonds represent significantly enriched KEGG pathways

In order to understand the molecular regulatory mechanism of lncRNA underlying the regulation of the immune function in subclinical mastitic cows fed or supplemented with folic acid, the target genes of DE lncRNAs were predicted. LncRNAs can regulate the protein-coding genes adjacent to their coordinates, so the mRNAs adjacent to lncRNAs were selected as their *cis*-target genes. In the SF vs. SC comparison group, a total of 523 mRNAs were found located within the 100 kb upstream and downstream of 84 DE lncRNAs, including 24 DE mRNAs (Table S9). Whereas a total of 333 mRNAs, including 22 DE mRNAs, were found located within 100 kb upstream and downstream of 55 DE lncRNAs in the comparison of HF vs. HC (Table S10). Only one novel gene (*ENSBTAG00000006383)* was shared by the two comparisons. In addition, we observed 10 DE protein-coding genes were very close (± 1 kb ) to seven DE lncRNA genes, including *COL4A2*, *GBP2*, *KIR3DL2* and other genes in the comparison of SF vs. SC. In order to find the lncRNA closely related to the phenotype (somatic cell count, SCC) in this study, the correlation analysis between the target gene expression of DE lncRNA and SCC found three genes *COL4A2* (Fig. 6A), *GBP2* (Fig. 6B), and *KIR3DL2* (Fig. 6C) were significantly related to SCC (*P* < 0.05).

The co-expression of lncRNAs and mRNAs was also performed to identify *trans*-target genes. A total of 84 DE lncRNAs were found to be significantly correlated with 104 DE mRNAs in the comparison of SF vs. SC (Pearson correlation, |R| > 0.95, *P* < 0.05). Meanwhile, 55 DE lncRNAs were significantly correlated with 132 DE mRNA in the comparison of HF vs. HC (Table S11, Table S12). Interestingly, we observed that the expression of some *cis*-target DE genes (e.g. *GBP2, CCL3, CCL4, KIR2DS1, PLPPR5, SGK1*) were also significantly correlated with the DE lncRNA. In this study, we predicted the sequence binding ability of *cis*- / *trans*-target genes and their corresponding lncRNAs. Using normalized free energy (ndG) < -0.08 as the screening standard, more than half (51.1%) of lncRNAs-target gens can be bound (ndG in the Table S9-12).

To explore the important pathways and biological functions of lncRNAs, KEGG pathway enrichment analysis and GO term analysis were performed on the target genes of DE lncRNAs. In the comparison of SF and SC, a total of 150 GO terms were significantly enriched (*P*-adjust < 0.01), and are found to be related to immune response functions, including cytokine activity, inflammatory response, response to the bacterium, cytokine receptor binding, immune system process, and so on (Fig. 6D and Table S13). Whereas, KEGG enrichment analysis revealed a total of 43 pathways were significantly enriched (*P*-adjust < 0.05, Table S14). Most of these pathways are related to immune or inflammatory responses. Among them, five DE genes (*CXCL3, ICAM1, GRO1, CCL3, CCL4*) were enriched in the above pathways, and they are the target genes of DE lncRNAs MSTRG.11108.1 and MSTRG.11828.1 (Fig. 6E).

### DE lncRNA and its target genes significantly enriched in somatic cell count- and mastitis-related QTLs

**Fig. 7:**
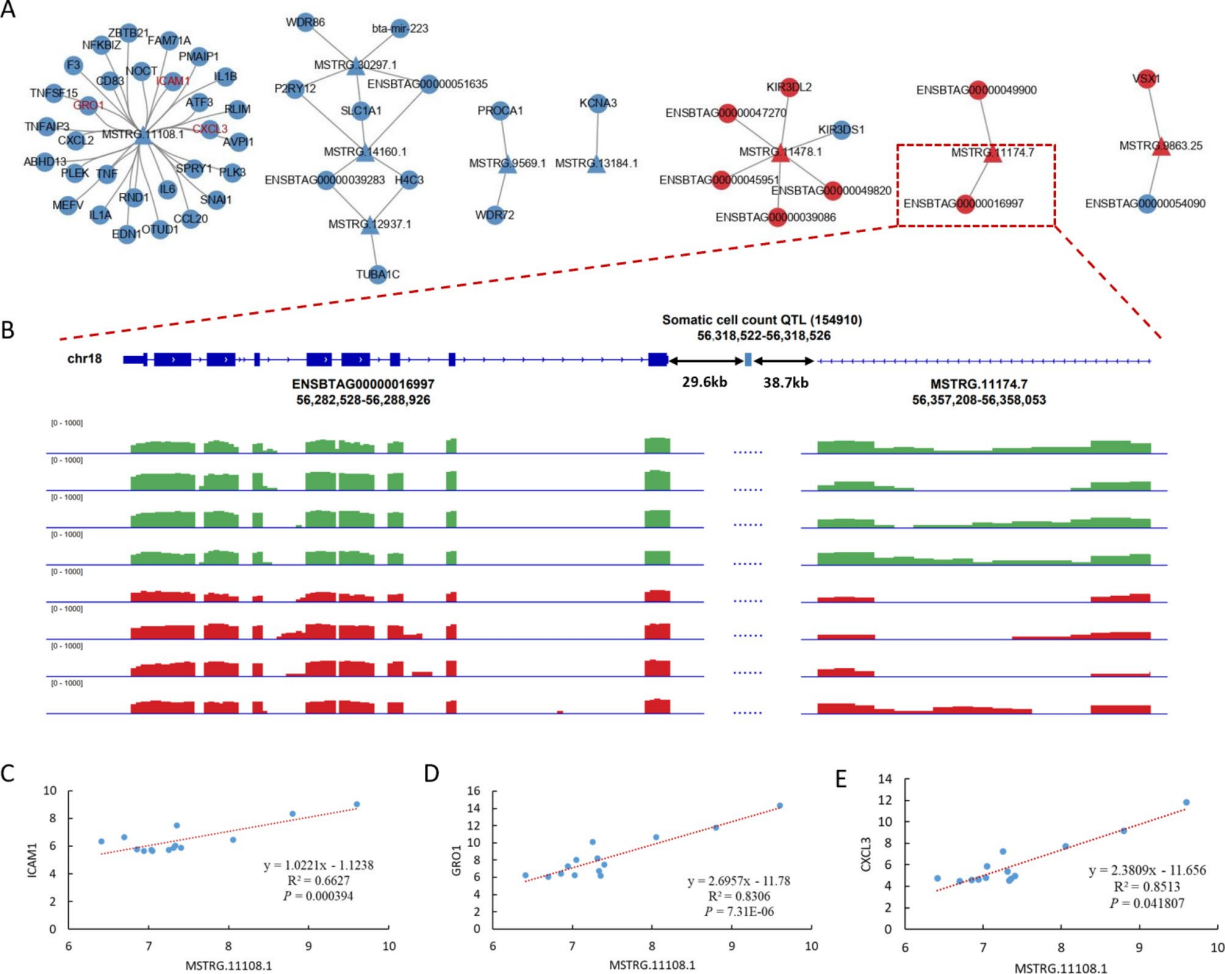
Integrate analysis with QTL data. (**A**) DE lncRNA and its DE target genes regulatory network enriched in QTLs of somatic cell score and clinical mastitis. (**B**) The positional relationship between the lncRNA MSTRG.11174.7, the target DEGs *ENSBTAG00000016997* and somatic cell count QTL (154,910). Green in the peak plot is the sample of SF group and red is the sample of SC group. The linear regression between (**C**) MSTRG.11108.1 and *ICAM1*, (**D**) MSTRG.11108.1 and *GRO1*, (**E**) MSTRG.11108.1 and *CXCL* using rlog-normalized read counts, respectively

**Fig. 8:**
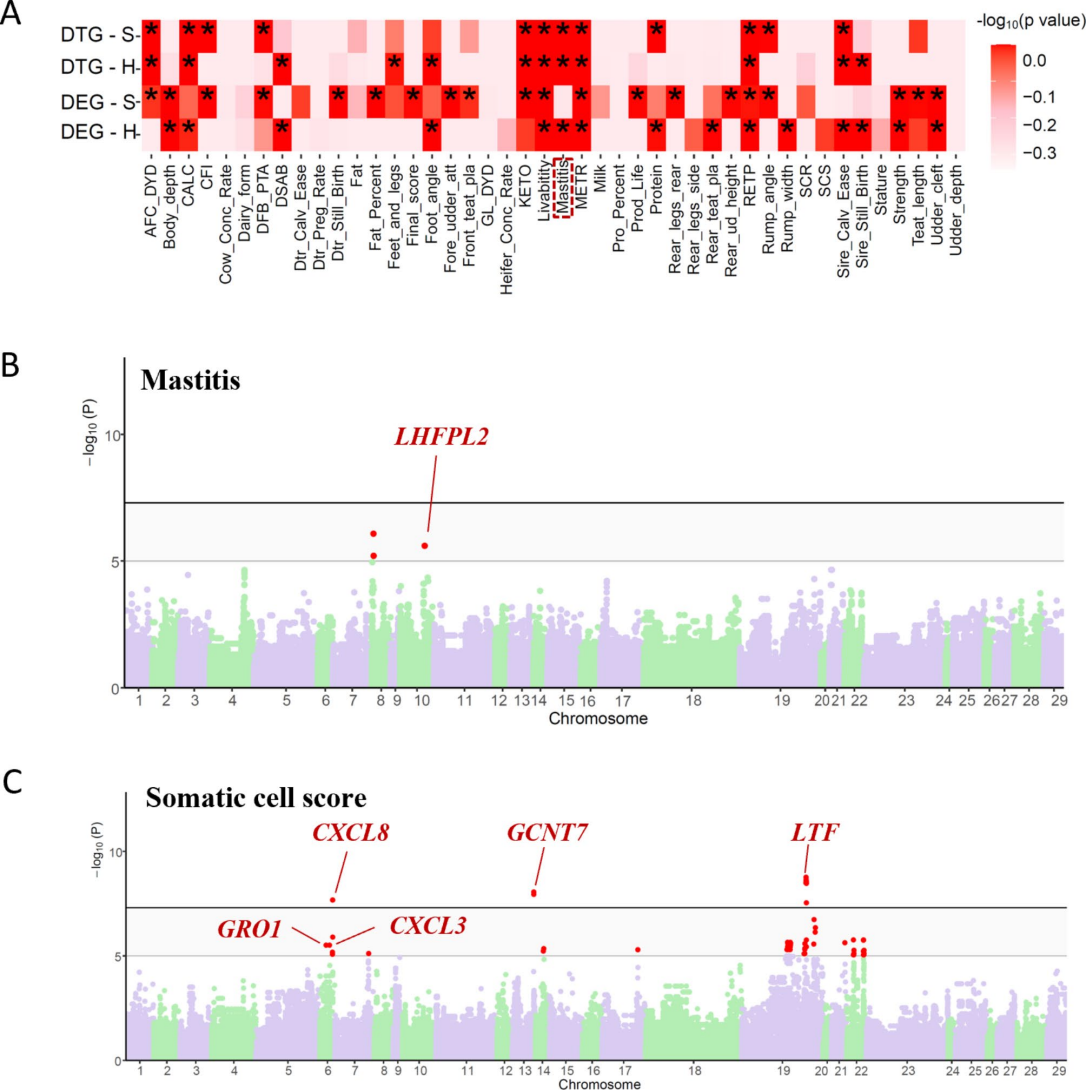
Integrate analysis with bovine GWAS data. (**A**) The relationships between 44 complex traits and four gene sets. The color corresponds to enrichment degrees [i.e., −log_10_(*P* + 1)] that are computed using a sum-based GWAS signal enrichment analysis based on the DEGs/DTGs and a 50 ± kb extension. (*) *P* < 0.05. DEG refers to differentially expressed genes; DTG refers to Target genes of DE lncRNA. Manhattan plot showing GWAS integration results in SF vs. SC (Genomic interval: 50 kb) for the trait of mastitis (**B**) and somatic cell score (**C**)

In order to better understand the relationship between lncRNAs in folic acid affecting subclinical mastitic cows, we selectively analyzed lncRNAs and their target mRNAs, among which lncRNAs and their neighbouring or expression-related genes were differentially expressed between the SF and SC groups. Based on the position of lncRNA in the genome, DE lncRNAs that overlap with 100 kb upstream and downstream of quantitative trait locus (QTL) were screened. By comparing the position of lncRNAs and QTLs, we found that 11 DE lncRNAs are distributed near 12 QTL regions of SCC/SCS/clinical mastitis (Table 3). The regulatory network of the above nine lncRNAs and their target DEGs is shown in Fig. 7A. For example, Fig. 7B shows the possible regulatory relationship between the lncRNA MSTRG.11174.7 and the target DEGs *ENSBTAG00000016997*. The distance of a related somatic cell count QTL (154,910) between them was also labeled. Among them, lncRNA MSTRG.11108.1 has the most target DEGs. The lncRNA MSTRG.11108.1 was significantly correlated with the target genes *ICAM1*, *GRO1* and *CXCL3* by regression analysis (Fig. 7C-E). This study used four sets of genes for GWAS integration analysis (Table 4). The results of GWAS enrichment analysis showed that SNPs related to mastitis were significantly enriched (Fig. 8A). Furthermore, the result also showed other health traits (Sire calving ease, metritis, ketosis) and production traits (e.g. milk protein) enriched by. Moreover, both DEGs and target genes of DE lncRNA can be significantly enriched by livability-related SNPs. For the trait of mastitis, 3 significant SNPs were enriched near *LHFPL2* (Fig. 8B). As shown in Fig. 8C, in the results of the target genes of DE lncRNA (DTG) in the comparison of SF vs. SC gene set, 108 significant SNPs for somatic cell score traits that were enriched near the DEGs *CXCL3*, *GRO1*, etc. For the DTG-HF vs. HC gene set, the results revealed that most of the significant SNPs are not adjacent to the differentially expressed genes. We summarized the details of all significant associations between traits and gene sets in the Table S15. The above-mentioned lncRNA MSTRG.11108.1 could be considered as a key lncRNA that plays a crucial role in the regulation of immunity in dairy cows with subclinical mastitis fed supplemented folic acid and warrants follow-up studies.

**Table 3:** The differentially expressed lncRNAs enriched in somatic cell score and clinical mastitis QTLs

chr	strart	end	transcript	strart	end	QTL
17	61,378,380	61,395,414	MSTRG.9569.1	61,449,556	61,449,560	Somatic cell score QTL (32,454)
17	71,135,959	71,143,749	MSTRG.9863.25	71,223,822	71,223,826	Somatic cell count QTL (154,834)
17	71,135,959	71,143,749	MSTRG.9863.25	71,238,170	71,238,174	Somatic cell count QTL (154,835)
18	55,476,038	55,476,403	MSTRG.11108.1	55,440,944	55,440,948	Somatic cell score QTL (49,858)
18	56,357,208	56,358,053	MSTRG.11174.7	56,318,522	56,318,526	Somatic cell count QTL (154,910)
18	62,625,500	62,714,586	MSTRG.11478.1	62,806,228	62,806,232	Somatic cell score QTL (49,936)
19	51,101,532	51,105,796	MSTRG.12937.1	51,070,944	51,070,948	Somatic cell score QTL (32,254)
19	56,816,326	56,846,862	MSTRG.13184.1	56,757,235	56,757,239	Clinical mastitis QTL (36,516)
2	106,176,194	106,184,237	MSTRG.14160.1	106,216,096	106,216,100	Clinical mastitis QTL (32,505)
5	30,135,703	30,140,607	MSTRG.24855.2	30,041,934	30,041,938	Clinical mastitis QTL (161,585)
5	43,266,426	43,268,842	MSTRG.25021.1	43,337,889	43,337,893	Somatic cell score QTL (175,697)
9	36,961,562	36,963,701	MSTRG.30297.1	37,050,843	37,050,847	Somatic cell score QTL (32,400)

**Table 4:** Four input gene sets used for GWAS integration analysis

Differential expression analysis	Differentially expressed genes (DEGs) *P* < 0.05, |FC| > 2		Target genes of DE lncRNA (DTGs) (± 100 kb or |R| > 0.95, *P* < 0.05)	
	Gene set name	Number of genes	Gene set name	Number of genes
SF vs. SC	DEG - S	215	DTG - S	1131
HF vs. HC	DEG - H	193	DTG - H	1529

## Discussions

In recent years, health traits including mastitic resistance have gradually been included in the breeding goals of the dairy industry [31–33]. The incidence rate of subclinical mastitis is high, and it is difficult to detect due to no obvious symptoms. Many studies have shown that folic acid can alleviate inflammatory reactions and improve immunity [28, 29], but its regulatory mechanism needs to be explored. At present, some studies show that lncRNAs play an important regulatory role in complex organisms (mastitis) [34–36]. Therefore, the effects of folic acid supplementation on subclinical mastitic and healthy cows, and the regulatory mechanisms of lncRNA on various immune-related genes (IRGs) were comprehensively compared and analyzed. The study revealed the key WGCNA modules, DEGs, DE lncRNAs, and the possible relation between the lncRNA expression and DEGs can be summarized in three findings.

This study analyzed and compared the transcriptome data of subclinical mastitic and healthy cows with and without folic acid supplementation. Folic acid is a water-soluble B vitamin (B9), which is closely related to metabolic pathways such as cell proliferation, milk protein synthesis and body immunity. It is an essential substance for maintaining the body’s normal life activities [37]. In recent years, with the rapid development of breeding and nutritional science, the production performance of dairy cows has rapidly improved. The folic acid synthesized by rumen microbes has been unable to meet the needs of high-yielding dairy cows [38]. Previous studies have shown that folic acid supplementation has a significant effect on immunity and lactation performance [28, 29, 39]. However, no significant changes in lactation performance were seen after supplementation of folic acid to subclinical mastitic cows [30]. In vitro, MAC-T cells were pre-treated with folic acid and then challenged with different strains of *Staphylococcus aureus*. It was found that folic acid plays a protective role in host defence against the *S. aureus* challenge partially. However, treated mac-T cells with different *S. aureus* strains and found that share a few differentially expressed and spliced genes, differentially expressed lncRNA, and activate different inflammatory responses in the host [40, 41]. The first finding of this study is that there are fewer common DE mRNAs and DE lncRNAs in the two comparisons of SF vs. SC and HF vs. HC. Although only a few of the same pathways were enriched in two comparisons (SF vs. SC and HF vs. HC), both of the comparisons were enriched by immune-related pathways. Folic acid supplementation affects the related inflammation and immune response pathways in subclinical mastitic dairy cows and improves metabolism and immune-related pathways in healthy dairy cows (Fig. 5A). For example, for the cell adhesion molecules (CAM) pathway shared by the two comparison groups. the DEGs enriched in CAM pathway are *ICAM*, *ITGA9*, etc. in SF vs. SC and *ICAM*, *JSP.1* in HF vs. HC. Based on this, it is speculated that this may be caused by the difference in the physical health status of subclinical mastitis cattle and healthy cattle. Subclinical mastitis is characterized by no clinical symptoms in milk and breasts, but physiological changes occur, such as SCC, sodium chloride content, pH value, and electrical conductivity [42]. Since subclinical mastitis in dairy cows is not easy to be detected, it causes long-term milk quality and milk production to decline. This may develop into clinical mastitis, increase the use of antibiotics and the elimination rate of dairy cows, and cause huge economic losses to the dairy industry [3, 43]. In general, although folic acid supplementation affects the immune-related pathways of dairy cows, there are some similar and different genes in the regulation process for subclinical mastitic cows and healthy cows.

The second finding is that lncRNA regulates gene expression through cis and trans-acting affecting phenotypes. Studies have shown that lncRNA regulates gene expression through cis- and trans-acting [44, 45]. The DE lncRNAs found in the present study were compared between the two comparisons (SF vs. SC and HF vs. HC) to establish whether or not folic acid supplementation significantly affected the expression of adjacent genes. In the comparison of SF vs. SC, 84 DE lncRNAs and 90 DEGs were significantly correlated in expression (*R* > 0.95, *P* < 0.05). Among these lncRNA-mRNA pairs, the locations of nine pairs are within 1 kb, including MSTRG.5075.2-*COL4A2*, MSTRG.22632.1-*GBP2*, MSTRG.11478.1- *KIR3DL2 etc. COL4A2* is coding for the alpha-2 chain of type IV collagen, an inhibitor of angiogenesis and tumor growth [46, 47]. A study carried out in breast cancer cell line MCF-7 showed that the reduction of *COL4A2* mRNA level may lead to increased invasiveness of MCF-7 cells [48]. Collagen is the main structural protein in the extracellular matrix (ECM) and an important regulator of the differentiation phenotype of breast epithelial cells in culture [49]. Specifically, the decrease of *COL4A2* level was related to the loss of basement membrane integrity, resulting in changes in the internal structure of the breast and affecting milk yield [50, 51]. Guanylate binding protein 2 (*GBP2*) is a member of the guanylate binding protein family (GBPs). Many pieces of evidence indicated that *GBP2* is an important participant in the host’s defence against intracellular pathogen infection [52, 53]. Studies on *GBP2* and tumors have found that *GBP2* is highly expressed in a variety of cancer patients and participates in the regulation of various regulatory factors during tumorigenesis [54, 55]. *KIR3DL2* is a gene that encodes Killer cell immunoglobulin-like receptors (KIRs) protein, which is a transmembrane glycoprotein expressed by natural killer cells and subsets of T cells. Among its related pathways are the Innate Immune System and Immunoregulatory interactions, which has the function of inhibiting cytotoxic [56]. In this study, compared with the SC group, *COL4A2* and *KIR3DL2* were significantly up-regulated, and *GBP2* was significantly down-regulated in the SF group (*P* < 0.05, log_2_|Fold Change| > 1). Although there is no direct example of folic acid in the above three genes, it is found that *COL4A2*, *KIR3DL2* and *GBP2* are significantly associated with SCC in this study (Fig. 6A-C). Therefore, it is speculated that folic acid regulates the expression of the above-mentioned genes by regulating lncRNA, and affects the body’s immune and inflammatory response processes.

The third finding is that several DE lncRNA and its target genes were significantly enriched in QTL/SNPs of mastitis and somatic cell count (SCC). Studies have shown that folic acid supplementation increases cell proliferation and reduces cell apoptosis [57]. This is consistent with the results of this study, enriching QTLs were related to bovine milk SCC or SCS. Combining with gene functions, pathways, QTL regions of SCS/SCC/mastitis, and GWAS results, candidate lncRNAs and their target genes related to folate-regulated immunity of cows with subclinical mastitis were identified, such as lncRNA MSTRG.11108.1 and its target genes *ICAM1*, *GRO1* and *CXCL3*. *ICAM1* encodes a cell surface glycoprotein which is typically expressed on endothelial cells and the cells of the immune system [58]. *ICAM1* plays an important role in promoting the adhesion of inflammatory sites, controlling tumor deterioration and metastasis, and regulating the body’s immune response [59]. *GRO1*, also known as *CXCL1*, is a member of the CXC subfamily of chemokines. Like *CXCL3*, *CXCL1* also plays a role in inflammation and as a chemoattractant for neutrophils [60–62]. The above results indicate that folic acid regulates the above-mentioned target genes through lncRNA MSTRG.11108.1 to improve immune response and suppress inflammation in cows with subclinical mastitis.

## Conclusions

The study uses RNA-Seq technology to screen differentially expressed lncRNAs in subclinical mastitic and healthy cows with folate supplementation. We combined our results with GWAS data and QTL information to provide more complete data for exploring the immune response mechanism of subclinical mastitic cows after folic acid supplement. The results of the study concluded that supplementary folic acid has a positive effect on the immunity of cows with subclinical mastitis and healthy cows. However, under different physiological conditions, the effects of folic acid supplementation on immunity are produced through different regulatory mechanisms. For subclinical mastitic cows, supplementation of coated folic acid can significantly affect the expression of immune-related pathway genes such as *ICAM1*, *GRO1* and *CXCL3*, while these genes’ expression may be regulated by lncRNA MSTRG.11108.1, thereby affecting related immune phenotypes. Since the role of lncRNA in subclinical mastitic cows has not been fully elucidated, our research provides a valuable starting point for future analysis. Therefore, this research lays a theoretical foundation for supplementing folic acid in subclinical mastitic cows in actual farm production management.

## Materials and methods

### Design of the study

In this study, a total of 111 Chinese Holstein cows with similar parities and body weight were used. All Chinese Holstein cows were obtained by Hebei Shounong Modern Agricultural Technology Co., Ltd. Then Subclinical mastitic cows were selected on the basis of the dairy herd improvement (DHI) data, The classification was based on the following criteria: cows whose somatic cell count (SCC) was more than 500 × 1000 cells / mL for two consecutive months without symptoms of clinical mastitis and were still in production herd as subclinical mastitic cows (high SCC cows). These cows were divided into the Subclinical mastitis supplementary feeding group (SF, *n* = 18), Subclinical mastitis control group (SC, *n* = 11), Health supplementary feeding group (HF, *n* = 40) and Health control group (HC, *n* = 42). The cows of SF and SC groups were bred at farm A in northern China and kept under standardized housing and feeding conditions. The experimental cows of HF and HC groups were provided by farm B in northern China.

### The dosage and plan of supplementary folic acid

Folic acid was purchased by Beijing Dongfang Tianhe Biotechnology Co., Ltd. and provided with a coating. The effective rate of coating folic acid on the rumen is 72%. According to the research conducted by Zhang[63] in the early stage, it was finally determined that the optimal dosage of coated folic acid in the experiment was 120 mg/500 kg, which was converted in proportion to body weight. For cows in SF and HF groups, the amount of folic acid was converted according to their body weight and mixed with concentrate. They were fed at the same time every day for 14 days. The two control groups (SC and HC) did not undergo any treatment.

### Sample Collection

After folic acid supplementation, 50 mL blood samples were collected from the caudal vein of each cow [64]. Then, peripheral blood leukocytes were collected by centrifugation at the speed of 3000 rpm for 15 min and stored in Trizol Reagent (Invitrogen, Carlsbad, CA, USA), placed in a liquid nitrogen container. Then immediately transferred to the laboratory, stored in the freezer at − 80℃ for further experiments.

### Total RNA extraction, library preparation, and transcriptome sequencing

Four samples were selected from SF and SC groups, and three samples from HF and HC groups for RNA extraction. Total RNA was extracted from the leukocytes sample using TRIzol (Invitrogen, Carlsbad, CA, United States) according to the manufacturer’s protocol. The concentration of total RNA was determined using the Nano 6000 spectrophotometer Assay Kit of the Bioanalyzer 2100 system (Agilent Technologies, Santa Clara, CA, United States). The RNA purity was determined using the Qubit® RNA Assay Kit in a Qubit® 2.0 Fluorometer (Life Technologies, Camarillo, CA, United States). The contamination and degradation of RNA quality were checked on 1% agarose gel electrophoresis. The RNA integrity number (RIN) and the OD260/280 of all samples were > 8.0, and > 1.8 respectively and were good enough to carry out the sequencing.

Sequencing libraries were constructed with the NEBNext® Ultra™ RNA Library Prep Kit for Illumina® (NEB, Ipswich, MA, USA) following the manufacturer’s protocol. Finally, the RNA libraries were sequenced on Illumina NovaSeq 6000 System by Annoroad Gene Technology Co., Ltd. and generated 150 bp paired-end reads.

### Quality control, alignment, and transcriptome assembly

For raw sequencing reads, FastQC_v.0.11.9 (http://www.bioinformatics.babraham.ac.uk/projects/fastqc/) was used to evaluate the quality. Quality trimming was performed with NGSQCToolkit_v2.3.3[65] by removing adapter molecules, reads containing poly-N, and low-quality reads, and then obtained clean data for subsequent analysis. Sequencing reads were mapped to the bovine genome (Ensembl annotation release 102, Bos_taurus.ARS-UCD1.2.102.gtf) using HISAT2_v.2.1.0[66] with default settings. After alignment, SAMtools_v.1.11[67] was used to sort the generated SAM files into BAM files. In all samples, mapped reads were assembled with Stringtie_v.1.3.4[68] based on the reference genome. Then, all assembled files are constructed into a new merged annotation file. Finally, assembled transcripts were annotated by gffcompare_v. 0.12.1[69].

### Identification of putative lncRNA

According to the structural and functional characteristics of lncRNA, the following filtering pipelines (Fig. 1) were employed to obtain putative lncRNA:1) Transcripts with exon number ≥ 2 were reserved from assembled transcripts; 2) Transcripts with read-count > 0 and length ≥ 200 nt were retained; 3) Based on class code only transcripts with class codes ‘i’, ‘u’, and ‘x’ (i: transcripts of intron region; u: transcripts of the intergenic region; x: Antisense transcripts of the exon) were selected; 4) Four different software were used in the prediction of protein-coding potential of the transcripts, namely, PLEK (predictor of long non-coding RNAs and messenger RNAs based on an improved k-mer scheme), CPAT (Coding Potential Assessment Tool), CNCI (Coding-Non-Coding-Index) and CPC2 (Coding Potential Calculator 2). The PLEK score < 0, CPAT Coding Label = No, CNCI score < 0, and CPC2 score < 0 were used as criteria for selecting non-coding transcripts which were used as the putative lncRNAs for subsequent analysis. Transcripts with class code ‘=’ were considered as known lncRNAs and also used in further analysis. The non-coding transcripts selected from the four software tools were employed as input to draw the Venn diagram (Fig. 2A). Then the transcripts which were at the intersection of the four software tools were selected for subsequent analyses.

### Fragment counting and differential expression analysis

According to the number of fragments or reads, the expression abundance of lncRNA and mRNA was calculated by HTSeq-count[70] under the default setting. Genes were filtered for read counts ≥ 10 in at least two samples. Because of the difference in library size and sequencing depth, we need to normalise read counts or fragment counts. DESeq2 R package[71] was used for obtaining the rlog-normalized read counts and the differential expression analysis. The statistical significance threshold for differentially expressed genes(mRNA or lncRNA)was set to *P-value* < 0.05 [Benjamini-Hochberg(BH) test] and log_2_ |Fold Change| > 1(detected by DESeq2).

### Weighted gene co-expression network analysis

In order to combine gene expression with phenotypic data for analysis, we performed co-expression analyses. Weighted gene co-expression network analysis (WGCNA) of all the protein-coding genes detected in this study was performed by the WGCNA R package[72]. The co-expressed genes within the WGCNA were identified as gene network modules (GNMs) marked with different colors. Highly interconnected genes within GNMs are co-regulated and might be involved in similar biological pathways. The module characteristic gene (ME) of the module is associated with the phenotype matrix to obtain the correlation and P value between each module and different physiological processes. Finally, *P* < 0.05 was used as the significance threshold, and the GNMs related to the phenotype were screened out.

### Target genes prediction of DE lncRNA

The biological function of lncRNA was predicted by studying the position (*cis*) and expression correlation (*trans*) between lncRNA and protein-coding genes. The prediction principle of *cis* target genes is that lncRNA affects its adjacent target genes. The protein-coding genes located 100 kb upstream and downstream of lncRNA were defined as *cis* target genes. *Trans* target genes were performed by calculating the correlation between lncRNA and mRNA expression levels. For the identification protein-coding genes, Pearson correlation coefficient (PCC) > 0.95 and *P-value* < 0.05 between DE-lncRNAs and DE-mRNA were calculated using custom scripts. The software LncTar was used to predict the binding ability of DE lncRNA and target genes [73]. The smaller normalized free energy (ndG) between sequences, the easier it is to bind. Generally, ndG < − 0.08 is selected as the criterion for binding.

### GO and KEGG enrichment analysis

KEGG (Kyoto Encyclopedia of Genes and Genomes) [74] is a public database that can be used to identify the main biological processes and signalling pathways enriched by the genes. GO (Gene Ontology) [75] clusters genes according to their biological processes (BP), cellular components (CC) and molecular functions (MF). In this study, All DE lncRNAs’ target genes and DE mRNAs were subjected to GO and KEGG to explore the potential biological functions. Additionally, KEGG analysis was also performed on genes in GNMs significantly related to phenotype. The above-mentioned GO and KEGG enrichment analyses were performed by the WebGestalt (WEB-based GEne SeT AnaLysis Toolkit, http://www.webgestalt.org/) [76, 77].

### Integrate analysis with QTL and GWAS data

The cattle QTLs related to clinical mastitis, somatic cell count (SCC), and somatic cell score (SCS) were extracted from the animal QTL database (https://www.animalgenome.org/cgi-bin/QTLdb/index/). According to the location information, differentially expressed lncRNAs and their target genes were compared and analyzed, the lncRNAs that had intersections with the above-mentioned QTLs were screened out. The significant SNPs for all traits of cattle were obtained from genome-wide association analysis (*n* = 27,143; https://figshare.com/s/ea726fa95a5bac158ac1) conducted by the University of Maryland and the U.S. Department of Agriculture (USDA). Finally, combined SNPs and lncRNA data for integrated analysis to determine whether the differentially expressed lncRNA or its target mRNA is significantly enriched in the GWAS signal (SNP) of complex traits (mastitis, etc.) in dairy cows.

### Electronic supplementary material

Below is the link to the electronic supplementary material.

Supplementary Material 1

Supplementary Material 2

## Data Availability

All raw and processed sequencing data generated in this study have been submitted to the NCBI BioProject under accession number PRJNA870216.

## References

[1] Vliegher SD, Fox LK, Piepers S, Mcdougall S, Barkema HW (2012). Invited review: Mastitis in dairy heifers: nature of the disease, potential impact, prevention, and control. J Dairy Sci.

[2] Barkema HW, Keyserlingk MA, Kastelic JP, Lam TJ, Luby C, Roy JP, LeBlanc SJ, Keefe GP, Kelton DF (2015). Invited review: changes in the dairy industry affecting dairy cattle health and welfare. J Dairy Sci.

[3] Gonalves JL, Kamphuis C, Martins CMMR, Barreiro JR, Tomazi T, Gameiro AH (2018). Bovine subclinical mastitis reduces milk yield and economic return. Livest Sci.

[4] Wang M, Liang Y, Ibeagha-Awemu EM, Li M, Mao Y (2020). Genome-wide DNA methylation analysis of mammary gland tissues from chinese holstein cows with *Staphylococcus aureus* induced mastitis[J]. Front Genet.

[5] Baloche G, Govignon-Gion A, Dassonneville R, Ducrocq V (2016). Multiple trait genetic evaluation of clinical mastitis in three dairy cattle breeds[J]. Animal.

[6] Urioste JI, Franzen J, Windig JJ, Strandberg E (2012). Genetic relationships among mastitis and alternative somatic cell count traits in the first 3 lactations of swedish Holsteins[J]. J Dairy Sci.

[7] Lichou F, Trynka G (2020). Functional studies of GWAS variants are gaining momentum[J]. Nat Commun.

[8] Qin Y, Sun W, Zhang H, Zhang P, Wang Z, Dong W, He L, Zhang T, Shao L, Zhang W (2018). LncRNA GAS8-AS1 inhibits cell proliferation through ATG5-mediated autophagy in papillary thyroid cancer[J]. Endocrine.

[9] Liu Y, Sun Z, Zhu J, Xiao B, Dong J, Li X (2017). LncRNA-TCONS_00034812 in cell proliferation and apoptosis of pulmonary artery smooth muscle cells and its mechanism[J]. J Cell Physiol.

[10] Touat Todeschini L, Shichino Y, Dangin M, Thierry Mieg N, Gilquin B, Hiriart E, Sachidanandam R, Lambert E, Brettschneider J, Reuter M. Selective termination of lncRNA transcription promotes heterochromatin silencing and cell differentiation[J]. EMBO J, 2017:e201796571. 10.15252/embj.201796571 PMC5579383 28765164

[11] Song G, Shen Y, Ruan Z, Li X, Chen Y, Yuan W, Ding X, Zhu L, Qian L (2016). LncRNA-uc.167 influences cell proliferation, apoptosis and differentiation of P19 cells by regulating Mef2c[J]. Gene.

[12] Liu W, Liu X, Luo M, Liu X, Luo Q, Tao H, Wu D, Lu S, Jin J, Zhao Y (2017). dNK derived IFN-γ mediates VSMC migration and apoptosis via the induction of LncRNA MEG3: a role in uterovascular transformation[J]. Placenta.

[13] Nan A, Jia Y, Li X, Liu M, Zhang N, Chen L, Yang T, Xu Y, Dai X, Cheng Y, editors. ‘s highlight:LncRNAL20992regulates apoptotic proteins to promote lead-induced neuronal apoptos[J]. Toxicol Sci, 2018,161(1):115–124. 10.1093/toxsci/kfx203 29029323

[14] Wang L, Zhao Y, Bao X, Zhu X, Kwok KY, Sun K, Chen X, Huang Y, Jauch R, Esteban MA (2015). LncRNA Dum interacts with dnmts to regulate Dppa2 expression during myogenic differentiation and muscle regeneration[J]. Cell Res.

[15] Wang J, Hua L, Chen J, Zhang J, Bai X, Gao B, Li C, Shi Z, Sheng W, Gao Y (2017). Identification and characterization of long non-coding RNAs in subcutaneous adipose tissue from castrated and intact full-sib pair Huainan male pigs[J]. BMC Genomics.

[16] Yu S, Zhao Y, Lai F, Chu M, Hao Y, Feng Y, Zhang H, Liu J, Cheng M, Li L. LncRNA as ceRNAs may be involved in lactation process[J]. Oncotarget, 2017,8(58). 10.18632/oncotarget.20439 PMC5716710 29228670

[17] Wang H, Cao Q, Ge J, Liu C, Ma Y, Meng Y, Wang Y, Zhao X, Liu R, Li C, Wang Y, Zhong J, Ju W, Jenkins EC, Brown WT, Zhong N. Lnc RNA -regulated infection and inflammation Pathways Associated with pregnancy loss: genome wide Differential expression of lnc RNA s in early spontaneous Abortion[J]. Am J Reprod Immunol, 2014,72(4). 10.1111/aji.12275 24916667

[18] Zhou M, Zhang Z, Zhao H, Bao S, Cheng L, Sun J (2017). An Immune-Related Six-lncRNA signature to improve prognosis prediction of Glioblastoma Multiforme[J]. Mol Neurobiol.

[19] Standaert L, Adriaens C, Radaelli E, Van Keymeulen A, Blanpain C, Hirose T, Nakagawa S, Marine J (2014). The long noncoding RNA Neat1 is required for mammary gland development and lactation[J]. RNA.

[20] Zhang Y, Xia J, Li Q, Yao Y, Eades G, Gernapudi R, Duru N, Kensler TW, Zhou Q (2014). NRF2/Long noncoding RNA ROR Signaling regulates mammary stem cell expansion and protects against Estrogen Genotoxicity[J]. J Biol Chem.

[21] Derakhshani H, Fehr KB, Sepehri S, Francoz D, Buck JD, Barkema HW, Plaizier JC, Khafipour E. Invited review: Microbiota of the bovine udder: contributing factors and potential implications for udder health and mastitis susceptibility[J]. J Dairy ence, 2018,101. 10.3168/jds.2018-14860 30292553

[22] Guéant JL, Namour F, Guéant-Rodriguez RM, Daval JL (2013). Folate and fetal programming: a play in epigenomics?[J]. Trends in Endocrinology & Metabolism Tem.

[23] Christensen KE, Qing W, Xiaoling W, Liyuan D, Caudill MA, Rima R (2010). Steatosis in mice is associated with gender, folate intake, and expression of genes of one-carbon metabolism[J]. J Nutr.

[24] Ly A, Lee H, Chen J, Sie KKY, Renlund R, Medline A, Sohn KJ, Croxford R, Thompson LU, Kim YI (2011). Effect of maternal and postweaning folic acid supplementation on mammary tumor risk in the Offspring[J]. Cancer Res.

[25] Ly A, Ishiguro L, Kim D, Im D, Kim S, Sohn K, Croxford R, Kim Y (2016). Maternal folic acid supplementation modulates DNA methylation and gene expression in the rat offspring in a gestation period-dependent and organ-specific manner[J]. J Nutr Biochem.

[26] Asaikkutti A, Bhavan PS, Vimala K (2016). Effects of different levels dietary folic acid on the growth performance, muscle composition, immune response and antioxidant capacity of freshwater prawn, Macrobrachium rosenbergii[J]. Aquaculture.

[27] Munyaka PM, Tactacan G, Jing M, House OK, Rodriguezlecompte JD (2012). Immunomodulation in young laying hens by dietary folic acid and acute immune responses after challenge with Escherichia coli lipopolysaccharide[J]. Poult Sci.

[28] Du HS, Wang C, Wu ZZ, Zhang GW, Liu Q, Guo G, Huo WJ, Zhang YL, Pei CX, Zhang SL (2019). Effects of rumen-protected folic acid and rumen‐protected sodium selenite supplementation on lactation performance, nutrient digestion, ruminal fermentation and blood metabolites in dairy cows[J]. J Sci Food Agric.

[29] Vanacker N, Girard CL, Blouin R, Lacasse P (2020). Effects of feed restriction and supplementary folic acid and vitamin B 12 on immune cell functions and blood cell populations in dairy cows[J]. Animal.

[30] Xueqin LIU, Di WANG, Siyuan MI, Ruiqing ZHANG, Liangyu SHI, Ying Y (2020). Molecular regulation mechanism of Key LncRNA in Subclinical Mastitis Cows with Folic Acid Supplement[J]. Acta Vet et Zootechnica Sinica.

[31] Miglior F, Fleming A, Malchiodi F, Brito LF, Martin P, Baes CF (2017). A 100-Year review: identification and genetic selection of economically important traits in dairy cattle[J]. J Dairy Sci.

[32] Weigel KA, Shook GE (2018). Genetic selection for Mastitis Resistance[J]. Veterinary Clin North Am Food Anim Pract.

[33] Gaddis K, Vanraden PM, Cole JB, Norman HD, Dürr JW. Symposium review: Development, implementation, and perspectives of health evaluations in the United States[J]. Journal of Dairy Science, 2020,103(6). 10.3168/jds.2019-17687 32331897

[34] Birney E, Stamatoyannopoulos JA, Dutta A, Guigó R, Gingeras TR, Margulies EH, Weng Z, Snyder M, Dermitzakis ET (2007). Identification and analysis of functional elements in 1% of the human genome by ENCODE pilot project[J]. Nature.

[35] Spizzo R, Almeida MI, Colombatti A, Calin GA (2012). Long non-coding RNAs and cancer: a new frontier of translational research?[J]. Oncogene.

[36] Yang J, Liu X, Wen T, Sun Y, Yu Y (2021). Progress on lncRNA regulated disease resistance traits in domesticated animals[J]. Hereditas.

[37] Ca Wley S, Mullaney L, Mckeating A, Farren M, Mc Ca Rtney D, Turner MJ (2016). A review of european guidelines on periconceptional folic acid supplementation[J]. Eur J Clin Nutr.

[38] Schwab EC, Schwab CG, Shaver RD, Girard CL, Putnam DE, Whitehouse NL (2006). Dietary forage and nonfiber carbohydrate contents influence B-vitamin intake, duodenal flow, and apparent ruminal synthesis in lactating dairy cows.[J]. J Dairy Sci.

[39] Zhang Z, La SK, Zhang GW, Du HS, Wu ZZ, Wang C, Liu Q, Guo G, Huo WJ, Zhang J. Diet supplementation of palm fat powder and coated folic acid on performance, energy balance, nutrient digestion, ruminal fermentation and blood metabolites of early lactation dairy cows[J]. Animal Feed Science and Technology; 2020. p. 265.

[40] Mi S, Tang Y, Shi L, Liu X, Si J, Yao Y, Augustino SMA, Fang L, Yu Y (2021). Protective roles of folic acid in the responses of bovine mammary epithelial cells to different virulent Staphylococcus aureus Strains[J]. Biology.

[41] Mi S, Tang Y, Dari G, Shi Y, Zhang J, Zhang H, Liu X, Liu Y, Tahir U, Yu Y. Transcriptome sequencing analysis for the identification of stable lncRNAs associated with bovine Staphylococcus aureus mastitis[J]. J Anim Sci Biotechnol, 2021,12(1). 10.1186/s40104-021-00639-2 PMC8667444 34895356

[42] Ruegg PL (2017). A 100-Year review: Mastitis detection, management, and prevention[J]. J Dairy Sci.

[43] Aghamohammadi M, Haine D, Kelton DF, Barkema HW, Hogeveen H, Keefe GP, Dufour S. Herd-level Mastitis-Associated costs on canadian dairy Farms[J]. Front Veterinary Sci, 2018,5. 10.3389/fvets.2018.00100 PMC5961536 29868620

[44] Guil S, Esteller M (2012). Cis-acting noncoding RNAs: friends and foes[J]. Nat Struct Mol Biol.

[45] Derrien T, Johnson R, Bussotti G, Tanzer A, Guigó R (2012). The GENCODE v7 catalog of human long noncoding RNAs: analysis of their gene structure, evolution, and expression[J]. Genome Res.

[46] Jeanne M, Gould DB. Genotype-phenotype correlations in pathology caused by collagen type IV alpha 1 and 2 mutations[J]. Matrix Biol, 2016:29. 10.1016/j.matbio.2016.10.003 PMC5328961 27794444

[47] Okada M, Yamawaki H (2019). A current perspective of canstatin, a fragment of type IV collagen alpha 2 chain[J]. J Pharmacol Sci.

[48] Wang C, Gao C, Zhuang JL, Ding C, Wang Y (2012). A combined approach identifies three mRNAs that are down-regulated by microRNA-29b and promote invasion ability in the breast cancer cell line MCF-7[J]. J Cancer Res Clin Oncol.

[49] Turashvili G, Bouchal J, Burkadze G, Kolar Z (2006). Wnt signaling pathway in mammary Gland Development and Carcinogenesis[J]. Pathobiology.

[50] Wang B, Sun H, Z NN, Zhu KJ, Liu JX. Amino acid utilization of lactating dairy cows when diets are changed from an alfalfa-based diet to cereal straw-based diets.[J]. Animal Feed Science & Technology; 2016.

[51] Wenting D, Quanjuan W, Fengqi Z, Jianxin L, Hongyun L (2018). Understanding the regulatory mechanisms of milk production using integrative transcriptomic and proteomic analyses: improving inefficient utilization of crop by-products as forage in dairy industry[J]. BMC Genomics.

[52] Praefcke, Gerrit JK. Regulation of innate immune functions by guanylate-binding proteins[J]. Int J Med Microbiol, 2017:S1084831811. 10.1016/j.ijmm.2017.10.013 29174633

[53] Kutsch M, Coers J (2021). Human guanylate binding proteins: nanomachines orchestrating host defense[J]. FEBS J.

[54] Wang Q, Wang X, Liang Q, Wang S, Xiwen L, Pan F, Chen H, Li D (2018). Distinct prognostic value of mRNA expression of guanylate-binding protein genes in skin cutaneous melanoma[J]. Oncol Lett.

[55] Yasutaka Y, Sho S, Takayuki A, Satoko K, Mayuko K, Atsushi O, Kazuto Y, Yukio N, Tomohiko I, Naohiko S (2018). Molecular pathogenesis of renal cell carcinoma: impact of the anti-tumor miR-29 family on gene regulation[J]. Int J Urol.

[56] Christian S, Anne MC, Armand B (2017). Therapeutic antibodies to KIR3DL2 and other Target Antigens on cutaneous T-Cell Lymphomas[J]. Front Immunol.

[57] Bae D, Chon JW, Kim DH, Kim H, Seo KH. Effect of folic acid supplementation on proliferation and apoptosis in bovine mammary epithelial (MAC-T) cells[J]. Animal Biotechnol, 2020(12):1–9. 10.1080/10495398.2020.1758123 32362185

[58] ICAM1 initiates (2021). CTC cluster formation and trans-endothelial migration in lung metastasis of breast cancer[J]. Nat Commun.

[59] Wei H, Wang Z, Kuang Y, Wu Z, Tong A. Intercellular adhesion Molecule-1 as target for CAR-T-Cell therapy of Triple-Negative breast Cancer[J]. Front Immunol, 2020,11. 10.3389/fimmu.2020.573823 PMC7539633 33072116

[60] Sharifi S, Pakdel A, Ebrahimi M, Reecy JM, Ebrahimie E (2018). Integration of machine learning and meta-analysis identifies the transcriptomic bio-signature of mastitis disease in cattle[J]. PLoS ONE.

[61] Huansheng H (2018). Identification of several key genes by microarray data analysis of bovine mammary gland epithelial cells challenged with Escherichia coli and Staphylococcus aureus.[J]. Gene.

[62] Islam MA, Takagi M, Fukuyama K, Komatsu R, Kitazawa H (2020). Transcriptome analysis of the inflammatory responses of bovine mammary epithelial cells: exploring Immunomodulatory Target genes for bovine Mastitis[J]. Pathogens.

[63] Zhichao Z. Effects of feeding folic acid on the early milk production performance of cows during perinatal period. China Agriculture University; 2016.

[64] Liu S, Yu Y, Zhang S, Cole JB, Tenesa A, Wang T, McDaneld TG, Ma L, Liu GE, Fang L (2020). Epigenomics and genotype-phenotype association analyses reveal conserved genetic architecture of complex traits in cattle and human[J]. BMC Biol.

[65] Patel RK, Mukesh J, Liu Z, NGS QC Toolkit. PLoS ONE. 2012;7(2):e30619. : A Toolkit for Quality Control of Next Generation Sequencing Data[J]. 10.1371/journal.pone.0030619 PMC3270013 22312429

[66] Kim D, Langmead B, Salzberg SL, HISAT. A fast spliced aligner with low memory requirements[J]. Nat Methods, 2015,12(4). 10.1038/nmeth.3317 PMC4655817 25751142

[67] Li H, Handsaker B, Wysoker A, Fennell T, Ruan J, Homer N, Marth G, Abecasis G, Durbin R (2009). The sequence Alignment/Map format and SAMtools[J]. Bioinformatics.

[68] Pertea M, Pertea GM, Antonescu CM, Chang TC, Mendell JT, Salzberg SL (2015). StringTie enables improved reconstruction of a transcriptome from RNA-seq reads[J]. Nat Biotechnol.

[69] Pertea M, Kim D, Pertea GM, Leek JT, Salzberg SL (2016). Transcript-level expression analysis of RNA-seq experiments with HISAT, StringTie and Ballgown[J]. Nat Protoc.

[70] Anders S, Pyl PT, Huber W. HTSeq - A Python framework to work with high-throughput sequencing data[J]. 2014. 10.1093/bioinformatics/btu638 PMC4287950 25260700

[71] Love MI, Huber W, Anders S (2014). Moderated estimation of fold change and dispersion for RNA-seq data with DESeq2[J]. Genome Biol.

[72] Langfelder P, Horvath S (2008). WGCNA: an R package for weighted correlation network analysis[J]. BMC Bioinformatics.

[73] Li J, Ma W, Zeng P, Wang J, Geng B, Yang J, Cui Q (2015). LncTar: a tool for predicting the RNA targets of long noncoding RNAs[J]. Brief Bioinform.

[74] Ogata H, Goto S, Sato K, Fujibuchi W, Kanehisa M (2000). KEGG: kyoto encyclopedia of genes and Genomes[J]. Nucleic Acids Res.

[75] Ashburner M, Ball CA, Blake JA, Botstein D, Butler H, Cherry JM, Davis AP, Dolinski K, Dwight SS, Eppig JT (2000). Gene Ontology: tool for the unification of biology[J]. Nat Genet.

[76] Wang J, Suhas V, Shi Z, Michael G, Zhang B. WebGestalt 2017: a more comprehensive, powerful, flexible and interactive gene set enrichment analysis toolkit[J]. Nucleic Acids Res, 2017(W1):W1. 10.1093/nar/gkx356 PMC5570149 28472511

[77] Liao Y, Wang J, Jaehnig EJ, Shi Z, Zhang B. WebGestalt 2019: gene set analysis toolkit with revamped UIs and APIs[J]. Nuclc Acids Research, 2019(W1):W1. 10.1093/nar/gkz401 PMC6602449 31114916

