# Supplementary material for: Molecular regulatory mechanism of key LncRNAs in subclinical mastitic cows with folic acid supplementation
Source: BMC Genomics. 2023 Aug 17;24:464. doi: 10.1186/s12864-023-09466-3 (PMC10436419; doi:10.1186/s12864-023-09466-3)
Supplement: Supplementary file 1 — Supplementary Material 1 [file 12864_2023_9466_MOESM1_ESM.docx]

**Supplementary Material**


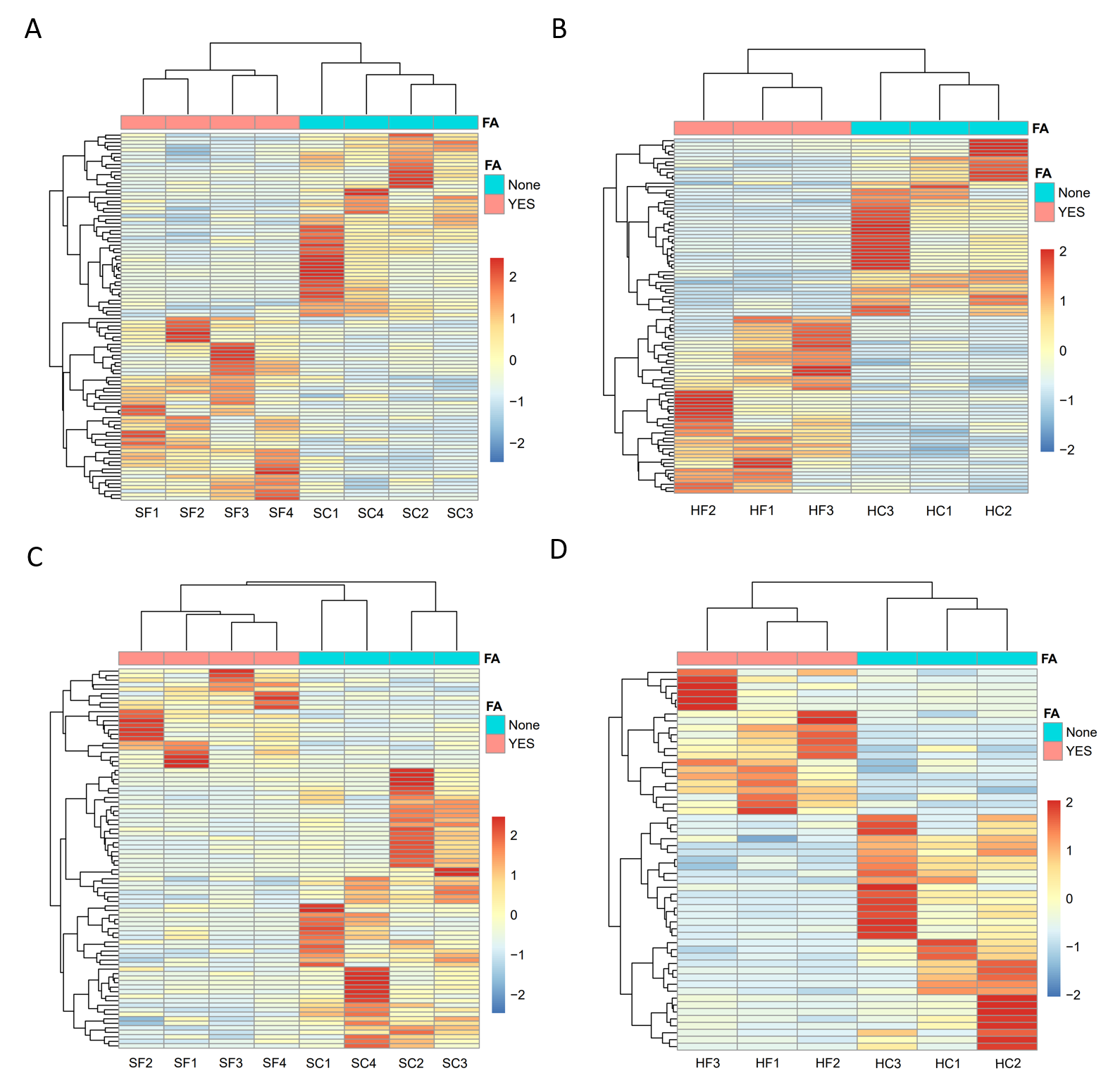


**Figure S1** The heatmap plots of the differentially expressed mRNAs and lncRNAs [log2(fold change) ≥1, P-value < 0.05]. Clustering analysis and heatmap of Upregulated Top 50 and downregulated Top 50 DE mRNAs in (A) SF vs. SC, (B) HF vs. HC; Clustering analysis of DE lnRNAs in (C) SF vs. SC, (D) HF vs. HC. Each row represents a gene, each column represents a sample, and color represents the level of gene expression (rlog-normalized read count) in the sample
